# Conformational Structures of Neutral and Cationic Pivaldehyde Revealed by IR-Resonant VUV-MATI Mass Spectroscopy

**DOI:** 10.3390/ijms232314777

**Published:** 2022-11-26

**Authors:** Sung Man Park, Yu Ran Lee, Chan Ho Kwon

**Affiliations:** 1 Department of Chemistry and Institute for Molecular Science and Fusion Technology, Kangwon National University, Chuncheon 24341, Republic of Korea; 2 Forensic Chemistry Division, National Forensic Service, Wonju 26460, Republic of Korea

**Keywords:** conformer-specific vibrational spectroscopy, photoionization, Franck–Condon analysis, trimethylacetaldehyde, VUV-MATI

## Abstract

Pivaldehyde, which is an unwanted by-product released with engine exhaust, has received considerable research attention because of its hydrocarbon oxidations at atmospheric temperature. To gain insight into the conformer-specific reaction dynamics, we investigated the conformational structures of the pivaldehyde molecule in neutral (S_0_) and cationic (D_0_) states using the recently invented IR-resonant VUV-MATI mass spectroscopy. Additionally, we constructed the two-dimensional potential energy surfaces (2D PESs) associated with the conformational transformations in the S_0_ and D_0_ states to deduce the conformations corresponding to the measured vibrational spectra. The 2D PESs indicated the presence of only the *eclipsed* conformation in the global minima of both states, unlike those in propanal and isobutanal. However, comparing the IR-dip VUV-MATI spectra from two intense peaks in the VUV-MATI spectrum with the anharmonic IR simulations revealed the correspondence between the *gauche* conformer on the S_0_ state and the measured IR spectra. Furthermore, Franck–Condon analysis confirmed that most peaks in the VUV-MATI spectrum are attributed to the adiabatic ionic transitions between the neutral *gauche* and cationic *eclipsed* conformers in pivaldehyde. Consequently, electron removal from the highest occupied molecular orbital, consisting of the nonbonding orbital of the oxygen atom in pivaldehyde, promoted the formyl-relevant modes in the induced cationic *eclipsed* conformer.

## 1. Introduction

Understanding the conformational structure of a polyatomic molecule is essential for elucidating the unique chemical reactivities of individual conformers and controlling their reaction dynamics, which has emerged as a research hotspot in chemistry [1,2,3,4,5]. However, deciphering the contribution of each conformation in the congested vibrational spectrum of the molecule is considerably challenging, because the conformers manifest comparable force fields between their atoms. Because of a low energy barrier, the conformers can be easily interconverted via rotation along a single bond between the carbons. Consequently, this process facilitates equilibration between all the conformers even at low temperature, resulting in limited conformer isolation. Therefore, the preparation of molecules with a specific conformation has been a persistent challenge [3]. Despite these difficulties, enabled by the technological advancements in ion spectroscopy, researchers have recently been successful in isolating a conformer in a molecule in exploring the conformer-specific photodissociation dynamics. For instance, Kim et al. observed conformer-specific photodissociation pathways for *gauche* and *anti* conformers in 1-iodopropane cation, which were directly generated using the vacuum ultraviolet mass-analyzed threshold ionization (VUV-MATI) spectroscopy [4]. In addition, Suits et al. combined photoelectron imaging with resonance-enhanced multiphoton ionization (REMPI-PEI) to elucidate the individual photodissociation dynamics for *cis* and *gauche* conformers in propanal cation [5]. Generally, these techniques involve adiabatic cooling of molecules, achieved via supersonic expansion, assuming the absence of the non-equilibrium kinetic effect [6]. Therefore, the molecular beam condition ensures that the molecules preferably populate the conformational and the vibrational ground states, because the populations depend on the Boltzmann distribution according to the vibrational temperature, which ultimately simplifies the conformational complexity.

In contrast, the REMPI-PEI technique is used to prepare the selected conformational or vibrational cations using multiphoton excitation via an intermediate state. Overall, an accurate characterization of the intermediate state is essential for selecting a molecular cation with a specific ionic state. However, in many non-aromatic molecular systems, the intermediate state either undergoes rapid internal conversion or is not clearly identified, thereby resulting in REMPI and corresponding photoelectron spectra with highly complicated features. In comparison, the VUV-MATI technique can generate molecular cations in either a specific conformation, vibrational state, or both, through direct transition from the neutral ground (S_0_) state to a Rydberg state converging to the ionic (D_0_) state without an intermediate state. As demonstrated, this technique facilitates the exploration of conformational structures of polyatomic cations by circumventing the structural complexity emerging from the veiled intermediate state in the multiphoton ionization process [7,8,9,10,11,12,13]. 

As such, the conformation corresponding to the peaks observed in the vibrational spectra can be identified by conducting Franck–Condon (FC) simulations based on the constructed potential energy surfaces associated with conformational interconversion. Similar investigations have revealed that molecules with sufficiently low energy barriers for conformational interconversion exist in thermal equilibrium even at low temperatures [7,8,10,11,12], whereas conformers with a high interconversion energy barrier retain their original composition during adiabatic cooling [9,13]. Thus, to understand the conformational stability of structures in the former case, further investigation is required on the conformational population depending on vibrational temperature. Nevertheless, deciphering the contribution of each conformer in the congested VUV-MATI spectrum of a polyatomic molecule is a challenging task owing to the presence of comparable force fields in the conformers. In this regard, certain alternative approaches, e.g., hole-burning and IR depletion spectroscopy methods, have been invented to characterize the structures of individual conformers [14,15]. In particular, these techniques exploit the principle that the excitation of a specific conformer in the ground state using a nanosecond pump laser irradiation can instantly reduce the population of its initial states. 

Moreover, the repopulation of the specific conformation in equilibrium promotes the development of various laser double-resonance spectroscopic techniques, including UV–UV hole burning, IR–UV hole burning, and IR ion-dip spectroscopies, which primarily focuses on the van der Waals complexes in the chromophore systems to adopt the REMPI scheme [16,17,18,19]. The subsequent efforts for analyzing the aliphatic molecules with extremely short lifetime in the excited electronic state motivated the development of the IR–VUV double-resonance techniques, despite the occasional emergence of uncontrollable fragmentations from excess VUV photon energy at 118 nm [20,21,22]. Extending the conventional laser double-resonance spectroscopic techniques, we recently developed a conformer-specific vibrational spectroscopy technique based on IR-resonant VUV-MATI mass spectroscopy, which can measure the identifiable vibrational spectra of individual conformers of a molecule in both S_0_ and D_0_ states [23]. As demonstrated, the newly invented techniques adopting the cold and collision-free environment of supersonic expansion can effectively elucidate the vibrational structures of individual conformers in both S_0_ and D_0_ states beyond the population redistribution [24]. 

Simple aliphatic aldehydes such as propanal and isobutanal, bearing one more methyl group on α-carbon, have garnered considerable research attention owing to their significance as key intermediates of molecular evolution in relevant astrochemical systems [25,26,27]. Since the stable conformations and the relative stabilities of these aldehydes are affected by formyl torsion, they have emerged as a stepping stone to understanding the astrochemical evolution of cold molecular clouds in peptide formation [8,10,28,29,30]. Suits et al. reported the conformational- and vibrational-selected photoion and photoelectron imaging studies of propanal [28] and isobutanal [30], wherein the adiabatic ionization energies (AIEs) were determined for each conformer in these aldehydes. The results suggested that the *gauche* conformer with CCCO dihedral angle of ~119° on the D_0_ state of propanal is slightly more stable—by only 65 cm^−1^—than the *cis* conformer with CCCO in planar, which reverses the conformational preference on the S_0_ state. Furthermore, they attributed a unique peak observed in the photoelectron spectrum of isobutanal to the *trans* conformer cation, which was less stable than the *gauche* conformer in the S_0_ state. 

The subsequent investigations regarding the AIEs and conformational structures of two aldehydes were performed by utilizing the VUV-MATI techniques adopting the one-photon scheme, which can directly investigate the intrinsic conformer-specific photoionization dynamics. Notably, the AIEs and cationic structures of the stable individual conformers in the propanal [29] and isobutanal [8,10] can be accurately deduced from the high-resolution (HR) VUV-MATI spectra. 

Kim et al. determined the AIEs of the *cis* and *gauche* conformers in propanal to be 9.9997 ± 0.0006 and 9.9516 ± 0.0006 eV, respectively, which produced the stabilization energy difference (94 cm^−1^) of the *gauche* and *cis* conformers in the D_0_ state. This finding was supported by the density functional theory (DFT) calculation for the S_0_ state that indicated the *cis* conformer as more stable (by ~294 cm^−1^) than the *gauche* conformer [29]. Lee et al. measured the VUV-MATI spectra of isobutanal under various molecular beam conditions involving several carrier gases and identified the peaks corresponding to the adiabatic ionic transitions of individual conformers (*gauche* and *trans*) with AIEs of 9.7398 ± 0.0004 eV and 9.6878 ± 0.0004 eV, respectively [8]. In addition, they accurately determined that the *gauche* conformer is more stable by 162 ± 50 cm^−1^ than the *trans* conformer in the S_0_ state, whereas the *trans* conformer is more stable by 262 ± 50 cm^−1^ than the *gauche* conformer in the D_0_ state. Subsequently, the HR conformer-specific vibrational spectrum of the cationic *gauche* conformer was acquired through conformationally effective cooling with Ar carrier gas. Based on the measured VUV-MATI spectrum of a pure *gauche* conformer, its cationic structure can be precisely determined by adopting the FC fitting method, which reveals that the promotions of the formyl torsion and CC stretching in the measured spectrum can be attributed to a unique ionization-induced transformation in the conformational structure. 

Investigating the effects of formyl torsion on the conformational structures and photoionization dynamics of pivaldehyde with three methyl groups on α-carbon will enable its comparative analyses with those of propanal and isobutanal. As pivaldehyde is emitted from vehicle exhausts and industrial sources associated with the oxidation of volatile organic compounds, it produces an acyl radical by the abstraction of the aldehydic H atom with OH radical, and therefore, is vital for atmospheric chemistry. More importantly, identifying the stable conformation of pivaldehyde is a prerequisite for understanding the conformer-specific reactivity at molecular level. Thus, to elucidate the conformational structures and their conformer-specific photoionization dynamics, this study performed IR-resonant VUV-MATI spectroscopy of pivaldehyde and constructed the two-dimensional potential energy surfaces (2D PESs) associated with the conformational transformations in the S_0_ and D_0_ states. Ultimately, this research aims to provide insights into conformational preference in terms of hyperconjugations between the methyl group(s) and formyl group in an aliphatic aldehyde.

## 2. Results and Discussion

### 2.1. Conformer-Specific Photoionization of Pivaldehyde 

**Figure 1:**
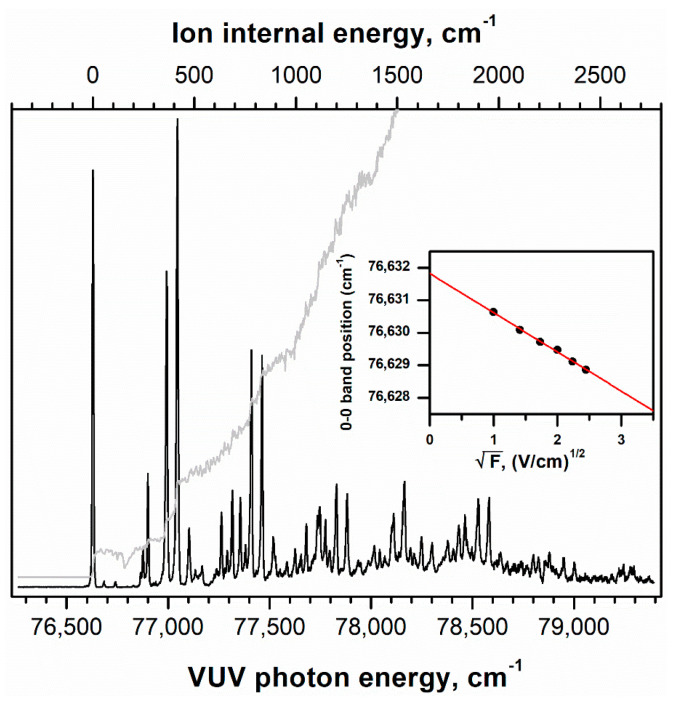
Delayed VUV–PIE curve (solid-gray line) for the C_5_H_10_O^+•^ signal measured as a function of photon energy of VUV laser pulse at the bottom horizontal axis. Based on the onset observed in the PIE curve, the HR VUV-MATI spectrum of pivaldehyde was recorded, where the 0–0 band position was located at 76,629 cm^−1^. Ion internal energy at the top horizontal axis corresponds to cationic vibrational frequency, estimated by setting the 0–0 band position as the origin. Inset: AIE of pivaldehyde determined accurately by extrapolating the 0–0 band position in the MATI spectrum to the zero-field limit.

To determine the ionization onset corresponding to the AIE value of the molecule, the delayed VUV-photoionization efficiency (PIE) curve of pivaldehyde was obtained by utilizing the pulsing scheme for the HR mode with the newly installed ion-source assembly [31]. The ionization onset was determined at ~76,626 cm^−1^, as denoted by the solid-gray line in Figure 1. Interestingly, several ascending steps appear above ~76,988 cm^−1^, indicating the possibilities of the onset of either another conformer, the favored FC transitions to low-frequency vibrational modes in the D_0_ state, as observed in the cases of propanal [29] and isobutanal [8], or both. To confirm these possibilities, the HR VUV-MATI spectrum of pivaldehyde was measured under the supersonic expansion condition (described in the Experimental Methods section) to eliminate any confusion with the hot bands and mostly populate the conformational and vibrational ground state, as illustrated in Figure 1. 

Apparently, the extremely intense peak appearing at 76,629 cm^−1^ in the VUV-MATI spectrum can be assigned to the 0–0 band of pivaldehyde, which corresponds to the adiabatic ionic transition between the neutral and cationic molecules in the vibrational ground states. However, upon ionizing the molecules in the zero-kinetic-energy (ZEKE) states situated at several cm^−1^ below the ionization threshold by the pulsed field ionization (PFI) field of 3.5 V cm^−1^ [31], the accurate AIE could be obtained by adjusting the PFI field to the zero-field limit, as depicted in the inset of Figure 1. Therefore, the AIE of pivaldehyde was determined to be 9.5012 ± 0.0004 eV (76,632 ± 3 cm^−1^), which is marginally higher but more accurate than the AIE value of 76,625 ± 6 cm^−1^ determined by one-photon MATI spectroscopy [32]. 

Notably, the AIE value determined herein is substantially less than the AIE values of the *gauche* and *trans* conformers in isobutanal [8], because the additional methyl group on the α-carbon of pivaldehyde is expected to increase the electron density of the formyl group.

**Figure 2:**
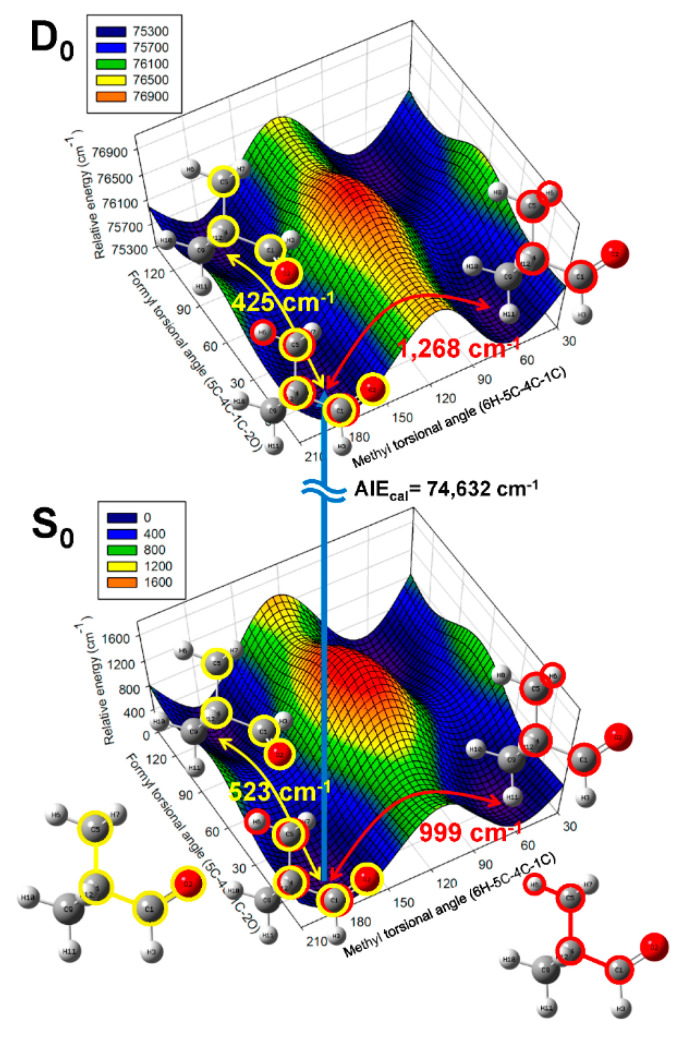
2D PESs of pivaldehyde in S_0_ and D_0_ states constructed as functions of dihedral angles associated with formyl and methyl torsional motions, describing conformational interconversion performed by optimizing the remaining geometric parameters at ωB97XD/cc-pVTZ level. Eclipsed-pivaldehyde conformer with C_S_ symmetry, which can be interconverted via three-fold internal rotations for formyl or methyl groups, exists at global minimum on both S_0_ and D_0_ states. AIE_cal_ denotes the AIE calculated for conformer with zero-point energy correction.

To ascertain information regarding the conformation corresponding to the measured AIE, the 2D PESs associated with the conformational variations occurring along the interconversion coordinates in the S_0_ and D_0_ states were constructed at the ωB97XD/cc-pVTZ level, as portrayed in Figure 2. The calculations were performed by optimizing the remaining geometric parameters, excluding two dihedral angles that were selected as the interconversion coordinates associated with the two torsional motions—formyl and methyl torsions—corresponding to the conformational interconversion of pivaldehyde. 

Unexpectedly, the constructed 2D PESs displayed that only the *eclipsed* conformer with C_S_ symmetry in pivaldehyde was located at the global minima on both states, whereas the staggered conformations such as *cis* or *trans* for propanal or isobutanal were located at the saddle point. Furthermore, the barrier height of 523 cm^−1^ for the formyl torsion in the S_0_ state, which is in agreement with the value of 385 cm^−1^ determined from microwave spectroscopy [33], was reduced to 425 cm^−1^ in the D_0_ state, implying that the formyl torsions in both states may involve a three-fold internal rotation potential. In contrast, the barrier height of 999 cm^−1^ in the S_0_ state for methyl torsion increased to 1268 cm^−1^ in the D_0_ state. The methyl torsions in both states can be described based on the harmonic potential instead of the internal rotation potential. This finding suggests that the variations in these barrier heights after ionization are primarily affected by the interactions between the staggered methyl hydrogens and the lone-pair electrons on the oxygen atom in the formyl group. This is further discussed based on comparison with the natural-bond orbital (NBO) analyses of the highest occupied molecular orbital (HOMO) and singly occupied molecular orbital (SOMO) for the neutral and cationic pivaldehyde. 

Overall, the measured AIE value corresponded to the adiabatic ionization transition between the neutral and cationic *eclipsed* conformers with C_S_ symmetry in the vibrational ground states. In addition, the AIE evaluated for the C_S_ conformation with zero-point energy correction was 74,632 cm^−1^, which is comparable to the measured AIE value, considering the employed calculation level. Therefore, most peaks appearing in the measured MATI spectrum corresponded to the vibrational modes of the cationic *eclipsed* conformer with C_S_ symmetry.

### 2.2. Conformational Structure of Neutral Pivaldehyde

**Figure 3:**
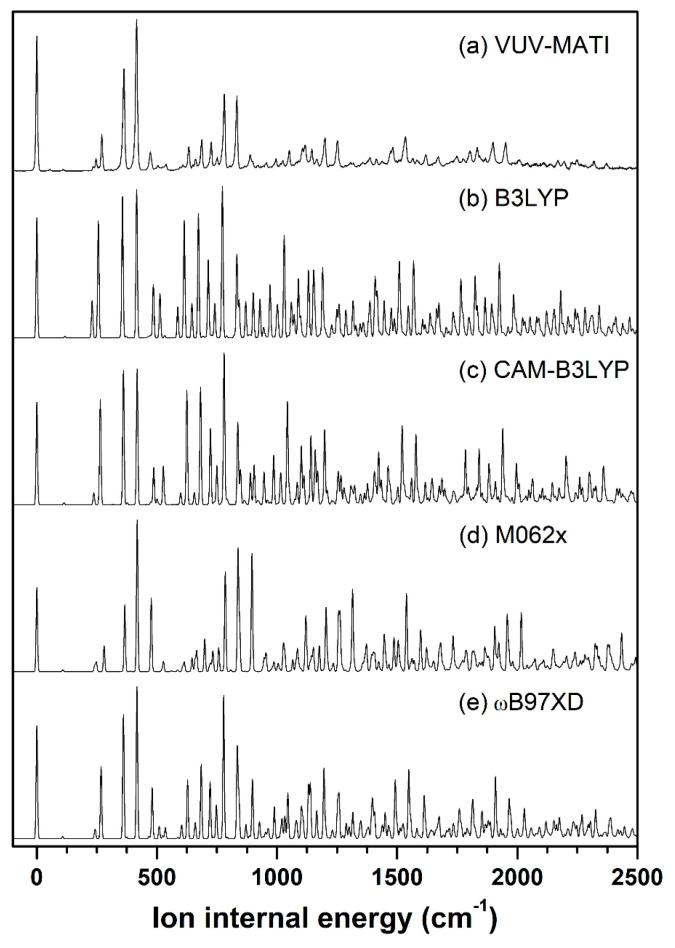
(**a**) HR VUV-MATI spectrum of pivaldehyde. Spectra simulated using FC factors and vibrational frequencies calculated at (**b**) B3LYP, (**c**) CAM-B3LYP, (**d**) M062X, and (**e**) ωB97XD levels with the cc-pVTZ basis set for adiabatic ionic transitions between optimized neutral and cationic pivaldehyde.

In general, the VUV-MATI spectrum essentially measures the adiabatic ionic transitions between the neutral and cationic states, and it is affected by the geometric variations induced upon ionization. With respect to the neutral geometry, the extent of variation in the cationic geometry can be determined using FC analysis. Thus, we evaluated the vibrational frequencies for neutral and cationic *eclipsed* conformers in pivaldehyde based on the geometries optimized at various density functionals with the cc-pVTZ basis set. Thereafter, the FC factors for adiabatic transitions from the vibrational ground state of the neutral to the individual vibrational states of the cation were calculated based on the evaluated normal-mode eigenvectors of the optimized geometries in both states. Moreover, the vibrational spectra of the cation simulated using the calculated FC factors and vibrational frequencies were compared with the VUV-MATI spectrum, as presented in Figure 3. At first glance, the entire simulated spectra shown in Figure 3b–e appear comparable to the VUV-MATI spectrum shown in Figure 3a, excluding the peaks with low internal energy of ions, corresponding to the cationic vibrational frequency estimated by selecting the 0–0 band position as origin. This comparability indicates the presence of a small vibronic coupling between the ion core and an electron in the high-lying Rydberg state. 

Furthermore, the measured MATI spectrum is related to the vibrational spectrum of the cationic *eclipsed* conformer, as predicted above. In particular, the FC factors and frequencies evaluated for the main adiabatic transitions in the simulated spectrum at ωB97XD/cc-pVTZ level (Figure 3e) were most comparable to their experimental counterparts for the prominent peaks located at 270, 363, 416, 632, 685, and 726 cm^−1^ in the MATI spectrum (Figure 3a). Accordingly, the frequencies and corresponding FC factors are listed in Table 1, including the ion internal energies of the experimental peaks, the intensities normalized by the most intense peak at 416 cm^−1^, and the frequencies of the neutral pivaldehyde determined by IR spectroscopy [34,35]. A careful comparison of these results implied that the FC factor was zero for the weak but distinct peak at 55 cm^−1^ on the MATI spectrum. This is because the related vibrational mode is due to formyl torsion, which is non-totally symmetric with C_S_ symmetry, and it facilitated transitions for the vibrations only with even quanta. In reality, all the formyl torsional vibrations appeared at 55, 110, and 166 cm^−1^ in the MATI spectrum. 

Moreover, other non-totally symmetric vibrations—under methyl torsion and bending for the cation with C_S_ symmetry—produced distinct peaks at 199 and 309 cm^−1^, respectively, in the measured spectrum. Generally, these appearances of non-totally symmetric vibrational modes for the cation may be caused by the breakdown of the C_S_ symmetry to the C_1_ symmetry. Surprisingly, the O–C–C–C dihedral angle between the experimental parameters of the neutral molecule was 2.1° (Table 2), determined using microwave spectroscopy [36], which was different from the calculated value of 0°; this indicates a *gauche* conformation. This finding implies that the appearances of the unexpected vibrational modes may be attributed to the inaccuracy in the neutral geometry calculated at the global minimum, unlike that in general cases [7,10,12,13], where the insufficient configuration interaction in the DFT calculations produces inaccuracies primarily in the cationic geometry of the open-shell system. 

To verify the inaccurate evaluation of the conformational geometry in the global minimum on the S_0_ state, the *eclipsed* and *gauche* geometries with 2O–1C–4C–5C dihedral angles of 0.0° and 2.1°, respectively, in the S_0_ state, were obtained by optimizing the remaining geometric parameters at the ωB97XD/cc-pVTZ level, as described in Table 2. As observed, all the geometric parameters evaluated for two conformations in the S_0_ state were consistent with the experimental results obtained using microwave spectroscopy [36], except for only the difference in the 2O–1C–4C–5C dihedral angle. Furthermore, the energy difference between the *eclipsed* and *gauche* geometries with zero-point energy correction was only 4 cm^−1^, implying that the inaccuracy propagated from the energy calculations of the stable conformation of the molecule [37].

**Figure 4:**
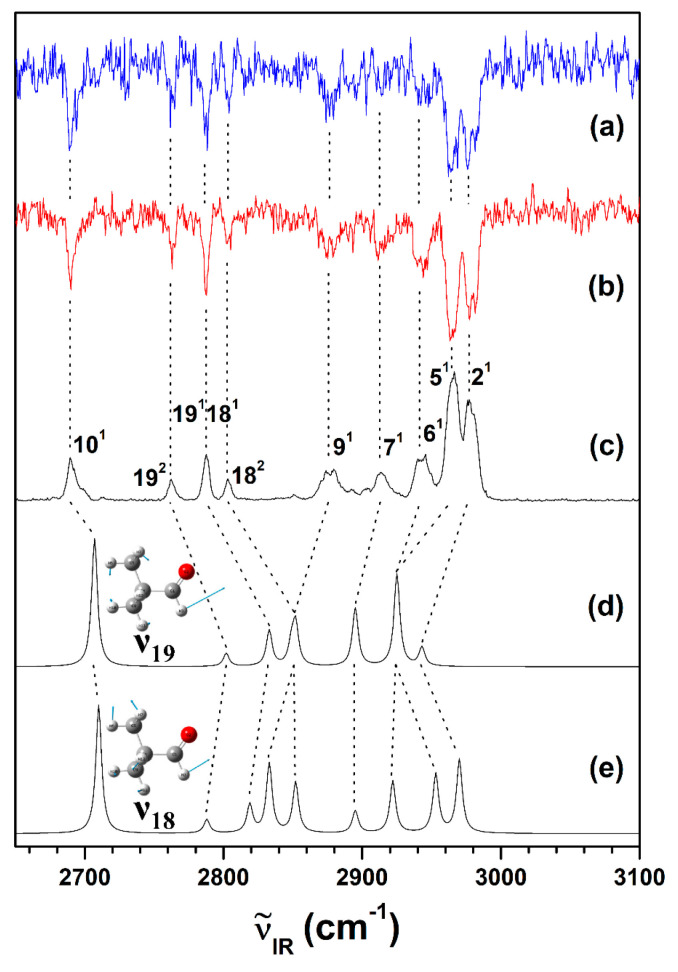
IR-dip VUV-MATI spectra of pivaldehyde were measured by monitoring intense peaks at (**a**) 76,629 and (**b**) 77,045 cm^−1^, respectively. (**c**) IR-resonant VUV-PI (76,529 cm^−1^) spectrum of pivaldehyde measured by scanning IR laser. Simulated IR spectra for neutral pivaldehydes with 2O–1C–4C–5C dihedral angles of (**d**) 0° and (**e**) 2.1°, respectively, calculated at ωB97XD/cc-pVTZ level with anharmonic potentials.

To investigate the abovementioned possibility utilizing the IR-resonant VUV-PI scheme, the IR absorption spectrum of gas-phase neutral pivaldehyde was obtained by scanning the frequency of the IR laser pulsed immediately before the VUV laser pulse fixed at 76,529 cm^−1^ below the ionization thresholds, as depicted in Figure 4c. More importantly, the conformer-specific vibrational spectra of the neutral pivaldehyde (i.e., the IR-dip VUV-MATI spectra) were recorded by monitoring two of the most intense peaks (76,629 and 77,045 cm^−1^) observed in the VUV-MATI spectra during scanning the frequency of the IR laser pulsating 5 ns in advance. This approach aimed to confirm that all the vibrational peaks observed in the measured IR absorption spectrum of pivaldehyde correspond to the single conformation, as discussed in the previous section. 

Notably, the two IR-dip VUV-MATI spectra (Figure 4a,b) were identical to the IR absorption spectrum (Figure 4c), and they were expected as a proxy for the vibrational spectrum of the stable conformer(s) in the neutral pivaldehyde, implying that the IR absorption and VUV-MATI spectra represent the vibrational spectra of only the single conformation in the neutral and cationic pivaldehyde, respectively. To inspect the symmetry between C_S_ (*eclipsed* conformation) and C_1_ (*gauche* conformation) of the neutral conformer, the IR spectra of the two conformations—*eclipsed* and *gauche*—in the S_0_ state were simulated with the harmonic vibrational frequencies calculated from two geometries at ωB97XD /cc-pVTZ level. However, the IR spectra of the two conformers (not presented in this study) varied from the experimental spectra (Figure 4a–c), because the Fermi resonance-related splitting peaks were observed as an aldehyde, and by borrowing the high intensity of the formyl stretching mode, either the overtones, the combination mode of the formyl bends, or in combination, appeared in the IR spectrum [38]. 

To represent Fermi resonance in the formyl stretching vibration region of pivaldehyde, the anharmonic vibrational frequencies were evaluated from the optimized *eclipsed* and the fitted *gauche* conformers in the S_0_ state and utilized in simulating the IR spectra, as depicted in Figure 4d,e, respectively. Remarkably, the IR spectrum simulated for the *gauche* conformer in neutral pivaldehyde (Figure 4e) is reasonably more comparable to the experimental IR spectrum than that (Figure 4d) for the *eclipsed* conformer, because 18^2^ and 9^1^ along with 6^1^ and 5^1^ can be identifiably distinguished in the calculated spectrum. Based on the simulated IR spectrum of the *gauche* conformer most consistent with the experimental spectra, the peaks observed in the IR spectra of the neutral pivaldehyde could be successfully assigned to the vibrational modes characterizing the formyl C–H stretching fundamental vibrations including the overtones and combinations of the formyl C-H bends along the vibrational frequencies measured by IR spectroscopy, as listed in Table 3 [34]. These findings revealed that the forbidden overtones and combination of the two formyl C-H bending modes (19 and 18) were promoted by the Fermi-resonance-related splitting with formyl C-H stretching vibration (10^1^).

### 2.3. Cationic Structure of Pivaldehyde 

**Figure 5:**
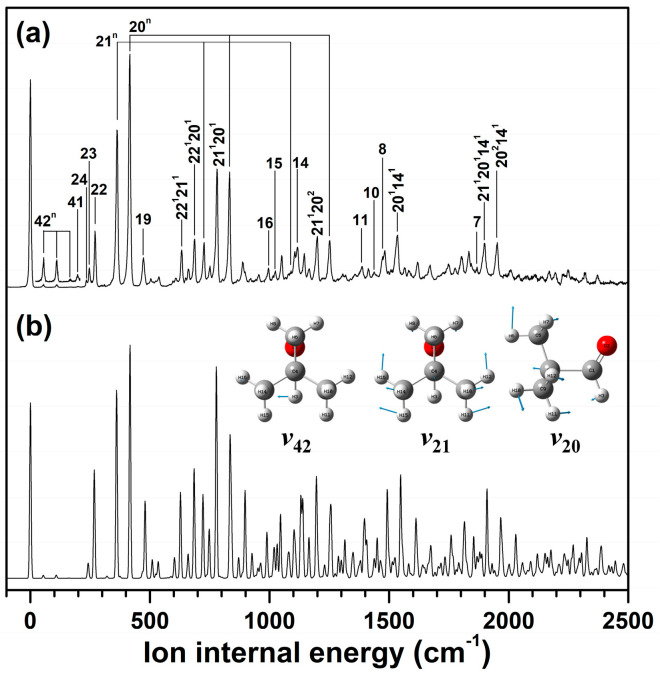
(**a**) HR VUV-MATI spectrum of pivaldehyde, including the vibrational assignments achieved by FC analysis. (**b**) FC simulation spectrum for adiabatic ionic transitions between neutral *gauche* with 2O–1C–4C–5C dihedral angle of 2.1° and the cationic *eclipsed* conformers optimized at ωB97XD/cc-pVTZ level for pivaldehyde. Eigenvectors of promoted vibrational modes in cationic state are illustrated in figure.

Observably, the measured VUV-MATI spectrum covered the adiabatic ionic transitions of the *gauche* conformer in the neutral pivaldehyde. Thus, for the individual vibronic transitions between the neutral *gauche* and cationic *eclipsed* conformers, the FC factors were estimated assuming the correct geometry in the D_0_ state. Thereafter, the vibrational spectrum of the cation was simulated based on the evaluated FC factors and the vibrational frequencies of the cation (Figure 5b) for comparison with the MATI spectrum (Figure 5a). As such, the simulated spectrum was comparable to the VUV-MATI spectrum in quantitative terms, implying that the cationic *eclipsed* conformer can be induced by the electron removal from the HOMO of the neutral *gauche* conformer. 

Accordingly, the calculated frequencies and corresponding FC factors for the cationic *eclipsed* conformer are listed in Table 1, including the vibrational frequencies corresponding to each peak in the MATI spectrum and the intensities normalized by the most intense peak at 416 cm^−1^. Based on the FC analysis with the simulated spectrum (Figure 5b), the vibrational assignment of the VUV-MATI spectrum of the *gauche* conformer in pivaldehyde could be accomplished. In brief, the intense peaks at 363 and 416 cm^−1^ with normalized intensities of 0.676 and 1.000 could be assigned to 21^1^ and 20^1^ related to the skeletal bends of the methyl groups incorporating the 1C–4C bond and 1C–4C–9(13)C bond angles, respectively. Moreover, these bond angles are consistent with their calculated frequencies (361 and 417 cm^−1^) and FC factors (0.815 and 1.000), respectively. These two strongly promoted vibrational modes can be attributed to the ionization-induced geometric variations and they produce overtones and combinations with other fundamental vibrational modes. Furthermore, the prominent vibrational peak at 270 cm^−1^ with an intensity of 0.242 was assigned to 22^1^, which is associated with the methyl torsional mode.

Based on rigorous vibrational assignment, these spectral features were caused by the elongation of the 1C–4C bond and the contractions of the 1C–4C–C_methyl_ and 2O–1C–4C bond angles as well as the 2O–1C–4C–5C dihedral angle in the cationic *eclipsed* conformation induced upon ionization with respect to the neutral *gauche* conformer. This is further discussed in the subsequent section from the perspective of NBO analysis. Accordingly, all the formyl torsional vibrations in the cationic state allowed in the MATI spectrum occurred due to the ionic transitions between the neutral *gauche* (C_1_ symmetry) and cationic *eclipsed* (C_S_ symmetry) conformers. The eigenvectors of the promoted vibrational modes in the cationic state are displayed in Figure 5, and the geometric parameters for the cationic *eclipsed* conformer are listed in Table 2.

### 2.4. HOMO and SOMO of Pivaldehyde 

**Figure 6:**
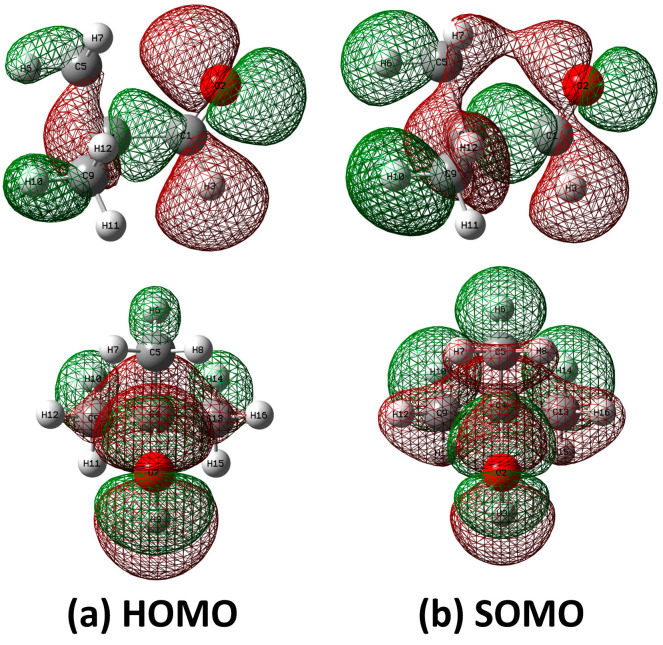
(**a**) HOMO of pivaldehyde with 2O–1C–4C–5C dihedral angle of 2.1° in S_0_ and (**b**) singly occupied molecular orbital (SOMO) of pivaldehyde in D_0_ state obtained at ωB97XD/cc-pVTZ level. HOMO and SOMO were obtained from NBO analysis.

The NBO analysis considers all interactions between the donor and acceptor NBOs to estimate the stabilization energy based on the hyperconjugation effect, which is closely related to the conformational preference in the S_0_ and D_0_ states. Accordingly, the NBO analyses on the HOMO of the neutral *gauche* conformer and SOMO of the cationic *eclipsed* conformer in pivaldehyde were performed at the ωB97XD/cc-pVTZ level. The results shown in Figure 6 indicate that the HOMO contains the lone-pair *p*-orbital on the oxygen atom of the formyl group interacting with σ-orbitals in the molecular plane. However, the SOMO generated by the adiabatic ionization of the neutral *gauche* conformer signified that the partially filled lone-pair *p*-orbital of the oxygen atom interacted with the σ-orbitals in the methyl group predominantly via the hyperconjugation effect to maximize charge delocalization. This created an *eclipsed* formyl group with a methyl group by altering the 2O–1C–4C–5C dihedral angle to zero. This phenomenon is similar to the NBO analysis results indicating the conformational preference between the cationic *gauche* and *trans* conformers in isobutanal [8]. 

Moreover, the vibrational modes related to the methyl and formyl torsional motions were commonly promoted after the ionization of the *gauche* pivaldehyde in the S_0_ state, which resulted from the charge-delocalized structure of the *eclipsed* pivaldehyde in the D_0_ state. Furthermore, this finding supports the fact that the adiabatic ionization-induced charge delocalization on the SOMO further diminishes the AIE of the *gauche* conformer in pivaldehyde compared to those in propanal and isobutanal, as discussed earlier.

## 3. Materials and Methods 

### 3.1. Experimental Methods

In this study, we performed IR-resonant VUV-MATI spectroscopy using the home-built HR VUV-MATI mass spectrometer, and its details were provided in previous studies [23,24,31,39].

Briefly, the coherent and tunable VUV light with a wavelength range of 126.0–131.1 nm was generated by resonant four-wave difference frequency mixing (FWDFM) in Kr-gas cell employing Kr 4p^6^–5p[1/2]_0_ or 4p^6^–5p[5/2]_2_ transitions, which were alternated to avoid wavevector mismatches with the VUV wavelength. For each transition, the required UV laser pulse at 212.556 or 216.667 nm with ~0.8 mJ/pulse was generated in a beta barium borate crystal, obtained via mixing after the doubling of 637.668 or 650.002 nm light, respectively, from a dye laser (Continuum, ND 6000) pumped by a Nd:YAG laser (Continuum, Surelite II). In addition, visible light with wavelengths ranging from 571.0 to 680.0 or from 623.0 to 700.2 nm with ~10 mJ/pulse from another Nd:YAG laser-pumped dye laser (Lambda Physik, Scanmate 2E) was spatially and temporally overlapped with 212.556-nm or 216.667-nm light. The combined beams were loosely focused in the Kr cell through a fused silica lens (f = 50 cm) to generate the tunable VUV laser pulse. Moreover, the pressure in the Kr cell was optimized at 4–6 Torr to maximize the generation of VUV laser pulses in the given photon energy range. Thereafter, the generated VUV laser pulse was spatially separated from the residual UV and VIS pulses by passing the beam off-center through a MgF_2_ lens at the exit of the Kr cell. Subsequently, it was introduced into the ion-source assembly in counter-propagation with the molecular beam.

Pivaldehyde was purchased from Sigma-Aldrich and used without further purification. From a reservoir at 298 K, the gaseous sample was supersonically expanded through a pulsed nozzle (diameter: 500 μm, Parker Valve) with the Ar stagnation pressure of 2–6 atm to ensure that most molecules populate the conformational and vibrational ground states [6]. To overlap with the VUV laser pulse within the new ion source, the generated molecular beam was introduced into the ionization chamber through a molecular beam skimmer (diameter: 1 mm, Beam Dynamics) placed ~3 cm downstream from the nozzle orifice. The background pressure in the ionization chamber was normally maintained at ~10^−7^ Torr when the nozzle was operated at 10 Hz. To remove the direct ions and electrons, a weak spoil field of ~0.2 V cm^−1^ was applied to the PFI stage. Simultaneously, a slight electric jitter—generated after an abrupt reduction in the high-voltage pulse—was used to increase the lifetime of the high-*n* Rydberg states via *m_l_* mixing, which yielded the ZEKE states [31].

Thereafter, the molecules in the ZEKE states, situated immediately below the ionization threshold conversing with the cationic states, were ionized and simultaneously accelerated into the delayed-extraction stage by a PFI field of 3.5 V cm^−1^ applied by the VUV laser pulse with a delay of 15 μs. Subsequently, the resulting MATI ions were extracted from the delayed-extraction stage for first-order space focusing. They passed through a field-free region and were subjected to mass-selective detection using the multichannel plates at the end of the TOF tube. The new electrode assembly and pulsing scheme adopted in the home-built VUV-MATI mass spectrometer could simultaneously enhance both the spectral resolution (~5 cm^−1^) and signal intensity of the measured VUV-MATI spectrum [31]. Furthermore, the intensity of each peak in the measured MATI spectrum was normalized to the power of the VIS laser pulse used for VUV generation by FWDFM.

For the IR-resonant VUV-PI/MATI scheme, HR IR laser pulse in the range of 2650–3100 cm^−1^ with ~7 mJ/pulse was generated using a narrowband optical parametric oscillator/amplifier (OPO/OPA) system (LaserVision, ~0.1 cm^−1^) pumped by a Nd:YAG laser (Continuum, Powerlite 8010) seeded with a wavelength of 1064 nm. Thereafter, the generated IR laser light was cylindrically focused using a telescope comprising two CaF_2_ lenses (f = 75 and −100 mm) aligned perpendicularly to both the molecular beam and the direction of ion flight in the photoionization chamber. The VUV laser pulse was delayed by 5 ns with respect to the IR laser pulse for photoexciting a specific conformer to a vibrational state. This extent of pulse delay was verified by accurately recording the IR-dip VUV-MATI spectrum of the target molecule, such that the relative delay of 5 ns between the IR and VUV laser pulses is adequate for repopulating a vibrational state in the high-frequency region. The frequencies of all lasers used in the experiments were measured and calibrated using a wavemeter (HighFinesse, Wavelength Meter WS5) with an accuracy of 0.1 cm^−1^.

### 3.2. Theoretical Methods

All the quantum chemical calculations were performed using the Gaussian 16 program package [40]. The levels of DFT, e.g., B3LYP, CAM-B3LYP, M062x, and ωB97XD, with the cc-pVTZ basis set afford reliable accuracy and were utilized to optimize the structures and frequencies of the pivaldehyde molecules in the S_0_ and D_0_ states. The equilibrium geometries on both the neutral and cationic states exhibited C_S_ symmetries. The IR spectra of the neutral *eclipsed* and *gauche* conformers in pivaldehyde were simulated at the ωB97XD/cc-pVTZ level with harmonic and anharmonic potentials for comparison with the experimental IR spectra. Subsequently, to analyze the MATI spectrum of pivaldehyde—essentially, the vibrational spectrum of the pivaldehyde cation—the FC factors were calculated based on the geometries, vibrational frequencies, and normal-mode eigenvectors of the neutral and cationic pivaldehyde in the ground electronic state at the ωB97XD/cc-pVTZ level. The vibrational frequencies calculated by harmonic approximation were appropriately scaled to compensate for the uncertainties arising from the vibrational anharmonicity and incomplete treatment of the electron correlation [41,42].

## 4. Conclusions

As the population of conformers in aliphatic aldehydes (e.g., propanal and isobutanal) depends on the vibrational temperature assuming adiabatic cooling under the molecular beam condition, the isolation and identification of a conformer are imperative. This study explored the conformational structures of pivaldehyde in the S_0_ and D_0_ states by utilizing conformer-specific vibrational spectroscopy.

The constructed 2D PESs associated with the conformational interconversion in the S_0_ and D_0_ states indicated the presence of only the *eclipsed* conformation at the global minima on both the states. In addition, we simulated IR spectra with anharmonic potentials for the neutral *eclipsed* and *gauche* conformations based on IR and microwave spectroscopies, respectively. The comparison of these simulated IR spectra with the observed IR-resonant VUV-PI/MATI spectra enabled the identification of the conformational structure of the neutral pivaldehyde in the ground electronic state and unveiled the *gauche* conformation of the neutral pivaldehyde.

Based on the conformation determined in the S_0_ state, the performed FC analysis could achieve the rigorous vibrational assignment of the measured VUV-MATI spectrum corresponding to the adiabatic ionic transitions between the neutral and cationic pivaldehyde, which indicated the cationic structure of the *eclipsed* conformer.

The ionization-induced geometric transformations can be attributed to the electron removal from the HOMO containing the lone-pair *p*-orbital on the oxygen atom that required charge delocalization via the hyperconjugation effect with the σ-orbitals in the methyl group in cationic state. Furthermore, this investigation of the conformational preference in the neutral and cationic states provides insights for elucidating the conformer-specific reaction dynamics upon photoionization and photodissociation in the gas phase.

## Figures and Tables

**Table 1:** Experiment and calculated vibrational frequencies (cm^−1^) of pivaldehyde in S_0_ and D_0_ states.

Mode ^a^	S_0_ (C_1_)		D_0_ (C_S_)					
			VUV-MATI		FC (C_S_→) ^d^		FC (C_1_→) ^c^	
	Exp ^b^	Cal ^c^	Freq	Int ^e^	Freq	Int ^e^	Freq	Int ^e^
**Fundamentals**								
42^1^	70	77	55	0.013	54		54	0.014
41^1^	222	196	199	0.004	195		195	1 × 10^−4^
39^1^	330	322	309	0.012	308		308	5 × 10^−4^
24^1^	267	250	235	0.031	239	0.012	239	0.013
23^1^	278	275	246	0.082	242	0.059	242	0.058
22^1^	348	346	270	0.242	267	0.475	267	0.472
21^1^	389	385	363	0.676	361	0.814	361	0.815
20^1^	593	590	416	1.000	417	1.000	417	1.000
19^1^	764	759	473	0.126	480	0.330	480	0.331
16^1^	1041	1038	996	0.081	989	0.069	989	0.069
15^1^	1217	1222	1025	0.070	1032	0.146	1032	0.147
14^1^	1277	1279	1117	0.171	1131	0.347	1131	0.347
11^1^	1406	1410	1387	0.089	1398	0.006	1398	0.006
10^1^	1457	1453	1437	0.067	1435	0.003	1435	0.003
8^1^	1484	1489	1473	0.132	1483	0.002	1483	0.002
7^1^	1774	1805	1867	0.086	1888	0.075	1888	0.074
**Overtones and combinations**								
42^2^	130	154	110	0.009	108	0.016	108	0.013
42^3^			166	0.001	162		162	0.001
24^1^22^1^			504	0.036	506	0.001	506	0.001
22^2^			538	0.045	535	0.069	535	0.067
24^1^21^1^			596	0.027	600	0.007	600	0.008
23^1^21^1^			610	0.039	603	0.086	603	0.085
22^1^21^1^			632	0.156	628	0.375	628	0.373
24^1^20^1^			650	0.039	656	0.011	656	0.013
23^1^20^1^			662	0.076	660	0.100	660	0.099
22^1^20^1^			685	0.196	685	0.476	685	0.472
21^2^			726	0.185	722	0.339	722	0.340
22^1^19^1^			750	0.081	748	0.213	748	0.211
21^1^20^1^			780	0.507	778	0.882	778	0.882
20^2^			833	0.494	835	0.579	835	0.578
20^1^19^1^			888	0.105	898	0.351	898	0.352
22^2^20^1^			955	0.054	952	0.036	952	0.035
22^2^19^1^			1015	0.050	1015	0.033	1015	0.032
22^1^21^1^20^1^			1051	0.135	1046	0.274	1046	0.272
21^3^			1090	0.070	1082	0.065	1082	0.066
22^1^21^1^19^1^			1106	0.153	1108	0.115	1108	0.115
21^2^20^1^			1145	0.144	1139	0.280	1139	0.280
21^1^20^2^			1200	0.220	1196	0.401	1196	0.400
20^3^			1251	0.199	1252	0.191	1252	0.191
20^2^19^1^			1306	0.057	1315	0.155	1315	0.155
21^1^16^1^			1357	0.057	1350	0.060	1450	0.060
20^1^16^1^			1413	0.008	1406	0.084	1406	0.084
21^1^14^1^			1482	0.157	1492	0.349	1492	0.349
19^1^15^1^			1496	0.068	1513	0.062	1513	0.063
20^1^14^1^			1536	0.225	1548	0.435	1548	0.435
21^2^20^1^19^1^			1619	0.108	1619	0.028	1619	0.028
21^1^20^2^19^1^			1671	0.095	1676	0.056	1676	0.056
22^1^21^1^14^1^			1748	0.099	1759	0.151	1759	0.150
23^1^20^1^14^1^			1776	0.082	1791	0.051	1791	0.051
22^1^20^1^14^1^			1803	0.131	1816	0.196	1816	0.194
20^2^16^1^			1833	0.154	1824	0.045	1824	0.044
21^2^14^1^			1843	0.102	1853	0.134	1853	0.134
21^1^20^1^14^1^			1899	0.188	1909	0.356	1909	0.356
20^2^14^1^			1952	0.191	1966	0.239	1966	0.238
20^1^19^1^14^1^			2008	0.075	2029	0.150	2029	0.150
23^1^20^2^14^1^			2195	0.064	2208	0.036	2208	0.036
20^3^16^1^			2248	0.075	2241	0.013	2241	0.013
21^1^20^2^14^1^			2320	0.061	2327	0.151	2327	0.150
14^1^8^1^			2591	0.041	2614	0.001	2614	0.001
19^1^15^1^14^1^			2614	0.049	2644	0.035	2644	0.035

^a^ Vibrational assignments for cationic pivaldehyde with C_S_ symmetry in Mülliken notation. ^b^ Frequencies from Refs. [34,35]. ^c^ Frequencies and FC factors calculated for the neutral with 2O–1C–4C–5C dihedral angle of 2.1° and cation with C_S_ symmetry at ωB97XD/cc-pVTZ level. The calculated vibrational frequencies were scaled by 0.975. ^d^ Frequencies and FC factors calculated for pivaldehyde with C_s_ symmetry in S_0_ and D_0_ states optimized at ωB97XD/cc-pVTZ level. ^e^ Normalized to intensity of the most intense peak at 416 cm^−1^ in MATI spectrum.

**Table 2:** Geometric parameters of pivaldehyde molecule in S_0_ and D_0_ states calculated at ωB97XD/cc-pVTZ level.

	S_0_			D_0_
	Exp ^a^	Cal (C_s_) ^b^	Cal (C_1_) ^c^	Cal (C_s_) ^b^
**Bond length (Å)**				
1C–2O	1.206	1.199	1.199	1.159 (−0.040) ^d^
1C–4C	1.516	1.516	1.516	1.724 (0.208)
4C–5C	1.537	1.523	1.523	1.505 (−0.018)
4C–9C (4C–13C)	1.537	1.535	1.535	1.515 (−0.020)
1C–3H	1.130	1.113	1.113	1.103 (−0.010)
5C–6H	1.118	1.091	1.091	1.093 (0.002)
5C–7H (5C–8H)	1.118	1.089	1.089	1.088 (−0.001)
9C–10H (13C–14H)	1.118	1.091	1.091	1.095 (0.004)
9C–11H (13C–15H)	1.118	1.092	1.092	1.089 (−0.003)
9C–12H (13C–16H)	1.118	1.091	1.091	1.089 (−0.002)
**Bond angle (°)**				
2O–1C–4C	126.0	125.9	125.9	121.0 (−4.9)
1C–4C–5C	110.5	110.9	110.8	108.3 (−2.5)
1C–4C–9C (1C–4C–13C)	107.4	107.1	106.9	100.5 (−6.4)
4C–1C–3H	113.0	113.8	113.8	110.6 (−3.2)
**Dihedral angle (°)**				
2O–1C–4C–5C	2.1	0.0	2.1	0.0 (−2.1)

^a^ Geometric parameters of neutral pivaldehyde determined using microwave spectroscopy in Ref. [33]. ^b^ Geometric parameters of neutral and cationic pivaldehyde calculated at ωB97XD/cc-pVTZ level. ^c^ Geometric parameters of neutral pivaldehyde with 2O–1C–4C–5C dihedral angle of 2.1° calculated at ωB97XD/cc-pVTZ level. ^d^ Numbers in parentheses represent ionization-induced geometric transformations with respect to the fitted *gauche* geometry.

**Table 3:** Vibrational assignment of pivaldehyde in high-frequency region (cm^−1^) of S_0_ state.

Mode (C_1_) ^a^	IR(C_S_) ^b^	Cal(C_S_) ^c^ (Har)	Cal(C_S_) ^d^ (Anhar)	Cal(C_1_) ^e^ (Anhar)	IR-Resonant VUV-PI	IR-Dip VUV-MATI	Mode Description ^f^
10^1^	2696	2727	2707	2710	2689	2689	C_formyl_-H stretch
19^2^	2768	2695	2802	2788	2762	2762	Overtone of C_formyl_-H bending
19^1^18^1^	2791	2722	2833	2819	2788	2788	Combination of bending
18^2^	2803	2750	2849	2833	2803	2803	Overtone of C_formyl_-H bending
9^1^	2876	2894	2852	2852	2876	2876	Out-of-plane (CH_3_)_2_ asym. stretch
7^1^	2913	2904	2895	2895	2913	2913	In-plane CH_3_ sym. stretch
6^1^				2922	2943	2943	Out-of-plane (CH_3_)_2_ asym. stretch
5^1^	2969	2964	2925	2953	2964	2964	Out-of-plane (CH_3_)_2_ sym. stretch
2^1^	2980	2976	2943	2970	2977	2977	In-plane CH_3_ asym. stretch

^a^ Vibrational assignments for neutral pivaldehyde with C_1_ symmetry in Mülliken notation. ^b^ Vibrational frequencies measured by IR spectroscopy in Ref. [34]. ^c^ Harmonic vibrational frequencies calculated from optimized geometry of neutral pivaldehyde with C_S_ symmetry at ωB97XD/cc-pVTZ level. The calculated vibrational frequencies were scaled by 0.951. ^d^ Anharmonic vibrational frequencies calculated for neutral pivaldehyde with C_S_ symmetry at ωB97XD/cc-pVTZ level to reflect Fermi resonances between the formyl C–H stretching and the overtone and combination of formyl C–H bends. ^e^ Anharmonic vibrational frequencies calculated for neutral pivaldehyde with 2O–1C–4C–5C dihedral angle of 2.1° at ωB97XD/cc-pVTZ level. ^f^ Normal modes for the optimized *eclipsed* conformer at the global minimum utilized for the vibrational assignments.

## Data Availability

The data that support the findings of this study are available from the corresponding author upon reasonable request.
